# T Cell Responses to the RTS,S/AS01_E_ and RTS,S/AS02_D_ Malaria Candidate Vaccines Administered According to Different Schedules to Ghanaian Children

**DOI:** 10.1371/journal.pone.0018891

**Published:** 2011-04-27

**Authors:** Daniel Ansong, Kwaku P. Asante, Johan Vekemans, Sandra K. Owusu, Ruth Owusu, Naana A. W. Brobby, David Dosoo, Alex Osei-Akoto, Kingsley Osei-Kwakye, Emmanuel Asafo-Adjei, Kwadwo O. Boahen, Justice Sylverken, George Adjei, David Sambian, Stephen Apanga, Kingsley Kayan, Michel H. Janssens, Marc J. J. Lievens, Aurelie C. Olivier, Erik Jongert, Patrice Dubois, Barbara M. Savarese, Joe Cohen, Sampson Antwi, Brian M. Greenwood, Jennifer A. Evans, Tsiri Agbenyega, Philippe J. Moris, Seth Owusu-Agyei

**Affiliations:** 1 School of Medical Sciences, Kwame Nkrumah University of Science and Technology, Kumasi, Ghana; 2 Kintampo Health Research Centre, Health Research Unit, Kintampo, Ghana; 3 London School of Hygiene and Tropical Medicine, London, United Kingdom; 4 GlaxoSmithKline Biologicals, Rixensart, Belgium; 5 Komfo Anokye Teaching Hospital, Kumasi, Ghana; 6 ImmunoVacc Consulting, Brussels, Belgium; 7 PATH Malaria Vaccine Initiative, Bethesda, Maryland, United States of America; 8 Kumasi Centre for Collaborative Research, Kumasi, Ghana; Agency for Science, Technology and Research - Singapore Immunology Network, Singapore

## Abstract

**Background:**

The *Plasmodium falciparum* pre-erythrocytic stage candidate vaccine RTS,S is being developed for protection of young children against malaria in sub-Saharan Africa. RTS,S formulated with the liposome based adjuvant AS01_E_ or the oil-in-water based adjuvant AS02_D_ induces *P. falciparum* circumsporozoite (CSP) antigen-specific antibody and T cell responses which have been associated with protection in the experimental malaria challenge model in adults.

**Methods:**

This study was designed to evaluate the safety and immunogenicity induced over a 19 month period by three vaccination schedules (0,1-, 0,1,2- and 0,1,7-month) of RTS,S/AS01_E_ and RTS,S/AS02_D_ in children aged 5–17 months in two research centers in Ghana. Control Rabies vaccine using the 0,1,2-month schedule was used in one of two study sites.

**Results:**

Whole blood antigen stimulation followed by intra-cellular cytokine staining showed RTS,S/AS01_E_ induced CSP specific CD4 T cells producing IL-2, TNF-α, and IFN-γ. Higher T cell responses were induced by a 0,1,7-month immunization schedule as compared with a 0,1- or 0,1,2-month schedule. RTS,S/AS01_E_ induced higher CD4 T cell responses as compared to RTS,S/AS02_D_ when given on a 0,1,7-month schedule.

**Conclusions:**

These findings support further Phase III evaluation of RTS,S/AS01_E_. The role of immune effectors and immunization schedules on vaccine protection are currently under evaluation.

**Trial Registration:**

ClinicalTrials.gov NCT00360230

## Introduction

Malaria, caused by the protozoan parasite *Plasmodium falciparum*, affects millions of people annually. Infants and young children in Africa carry most of the disease burden. *P. falciparum* has a complex life cycle including several developmental stages in its human host. The RTS,S malaria candidate vaccine, which has recently entered Phase III testing, targets the *P. falciparum* circumsporozoite protein (CSP), a pre-erythrocytic stage antigen. Implemented in the Expanded Program of Immunization (EPI), together with existing control measures such as wide spread use of insecticide treated nets, vector control and use of new generation anti-malaria drugs, RTS,S may contribute to sustained malaria control.

The vaccine antigen contains the central tandem repeats and carboxy-terminal regions of CSP fused to the N-terminal of hepatitis B virus surface antigen. Combination of this fusion protein with native hepatitis B surface antigen results in the spontaneous formation of RTS,S virus-like particles [1]. This antigen formulated with the AS02 adjuvant induces CSP specific adaptive immune responses and protection against infection in controlled parasite challenge studies [2]–[5] as well in semi-immune adults, children and infants living in malaria-endemic regions [6]–[11]. The AS02 adjuvant is based on an oil-in-water emulsion combined to the TLR4 ligand monophosporyl lipid A (MPL) and the QS21 saponin fraction of *Quillaja saponaria* [1]. A new RTS,S formulation containing the AS01 adjuvant and consisting of MPL, QS21 and liposomes was recently selected as an alternative to RTS,S/AS02 on the basis of its ability to induce comparable, or better, CSP-specific antibody responses and greater T cell responses in small animal models and non-human primates than did AS02 [12], [13]. This effect was confirmed in a Phase IIa controlled parasite challenge study performed in malaria naïve adults at the Walter Reed Army Institute of Research where, compared to RTS,S/AS02, RTS,S/AS01 was shown to be well tolerated, to induce strong humoral and cellular immune responses, and to improve protection against *P. falciparum* challenge [5]. Efficacy of RTS,S/AS01_E_ (the pediatric formulation of RTS,S/AS01) against malaria was then evaluated in children, with favourable results [14], and the possibility to safely co-administer RTS,S/AS01_E_ and EPI vaccines has been shown [15].

Both humoral and cellular immune responses play a key role in protection against *Plasmodium* infection in mice [16]–[21]. However, the relevance of these observations to the host-parasite relationship in humans remains to be demonstrated. Recent evidence indicates that, in adults, protection is associated with high titers of CSP-specific antibodies and CD4 T cell responses [5]. As pediatric populations are particularly susceptible to malaria, it is important to investigate humoral and cellular immune responses in a younger age group to provide a better understanding of the immune mechanisms which mediate protection following RTS,S vaccination.

The present study was designed to document the safety and immunogenicity of RTS,S/AS01_E_ and RTS, S/AS02_D_ (the pediatric formulation of RTS,S/AS02) in 5–17 month old children at two different sites in Ghana. Three schedules selected on the basis of compatibility with the existing EPI vaccination program were evaluated for induction of anti-CSP antibodies and T cell responses. Results of safety and humoral immunogenicity evaluations have been reported previously [22]. Briefly, both RTS,S/AS02_D_ and RTS,S/AS01_E_ were well tolerated and induced high titers of anti-CSP and anti-HBs antibodies. Recipients of RTS,S/AS01_E_ had higher peak anti-CSP antibody responses for all 3 schedules than did recipients of RTS,S/AS02_D_. Three-dose schedules induced higher antibody levels than 2-dose schedules. The peak antibody response following a 0,1,2-month schedule was higher than following a 0,1,7-month schedule, but area under the curve analyses of anti-CSP antibodies for the overall study period were comparable between the 0,1,2- and 0,1,7-month schedules for both vaccine formulations. T-cell responses, using whole blood antigen-stimulation followed by intracellular cytokine staining, are reported here.

## Materials and Methods

The supporting CONSORT checklist for this trial is available as supporting information; see Checklist S1. The protocol of this trial was posted with a previous publication (Protocol S1 [22]).

### Ethics statement

The protocol was approved by relevant ethical and institutional review boards as described previously [22]. The trial was undertaken according to the International Conference on Harmonization, Good Clinical Practice guidelines and was monitored by GlaxoSmithKline (GSK) Biologicals. The study was overseen by a formally constituted Data Safety Monitoring Board (DSMB) operating under a charter. Written informed consent was obtained from each child's parent(s) or guardian(s) before study procedures were initiated. Illiterate parents or guardians indicated consent using a thumbprint, and a signature was obtained from a literate witness.

### Study design and sampling

**Figure 1:**
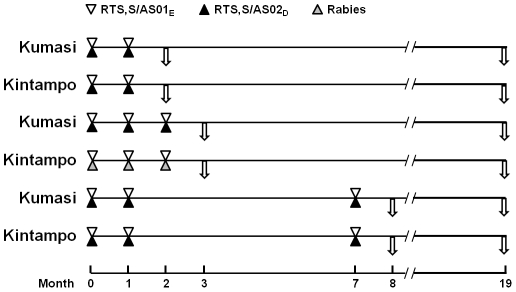
Schematic representation for evaluation of CSP specific T cell responses. Triangles represent timing of vaccination for 0,1-, 0,1,2- and 0,1,7-month schedules (RTS,S/AS01_E_, RTS,S/AS02_D_ or Rabies vaccine); arrows represent timing of blood sampling. In both research centers, each study group included 45 individuals.

The design of this trial [http://clinicaltrials.gov: NCT00360230] and CONSORT flowchart have been described in detail previously [22]. No changes were made to the protocol after ethical approval. Briefly, the trial was a Phase II randomized, controlled, partially-blind study. A total of 540 eligible subjects were randomly assigned to one of three study groups in each study site (Figure 1). In one site rabies vaccine (Purified chick embryo cell-culture rabies vaccine; Chiron Behring GmbH, Marburg, Germany), administered following a 0,1,2-month schedule, was used as a control. Blood samples for assessment of cell-mediated immune (CMI) responses were collected one month after the last vaccine dose in each vaccination schedule (peak response) and at month 19 of the study. The study was conducted in two locations in Ghana, about 200 km apart: one coordinated from Kumasi Centre for Collaborative Research/School of Medical Sciences (KCCR/SMS), Kumasi, with the field site in the town of Agogo and clinical activities centered at the Agogo Presbyterian Hospital, and the other one in the Kintampo area, with clinical activities centered at the Kintampo Health Research Centre (KHRC) and Kintampo Hospital. Malaria transmission in both areas is intense and perennial. Insecticide treated nets were distributed to potential study participants at screening.

### Detection of anti-CSP humoral responses

Serum antibodies to the NANP repeat region of CSP (B cell epitope) were measured by a standard, validated enzyme-linked immunosorbent assay (ELISA) using plates adsorbed with the recombinant antigen R32LR that contains the sequence [NVDP(NANP)152LR], at a GSK validated laboratory (CEVAC, University of Ghent, Belgium). Titers were calculated using a reference standard curve with a 4 parameter logistic fitting algorithm and expressed in EU/ml, with cut-off for seropositivity of 0.5 EU/ml [23].

### Whole blood intracellular cytokine staining and flow cytometry

**Figure 2:**
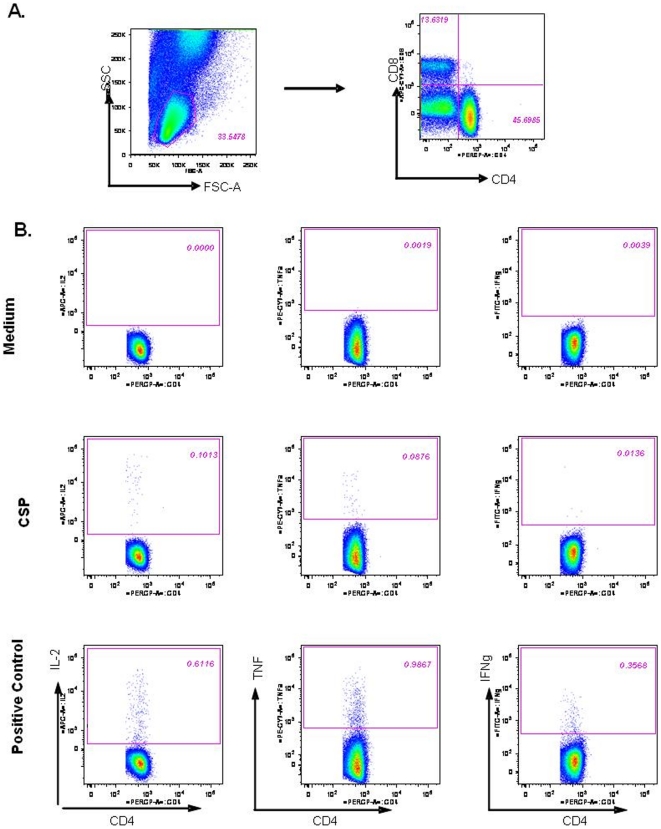
Whole-blood intracellular cytokine detection by flow cytometry. Whole-blood intracellular cytokine detection by flow cytometry was performed following overnight stimulation with medium (negative control), CSP and PHA (positive control). (A) CD4 or CD8 T cells were identified from a lymphocyte gate on an SSC-FSC plot. (B) IL-2^+^, TNF-α^+^, IFN-γ^+^, and CD40-ligand (not shown) CD4 T cells and CD8 T cells (not shown) were counted. The unstimulated sample (medium) shows background levels of cytokine production, while the stimulation with PHA (positive control) shows strong production of IL-2, TNF-α, or IFN-γ by CD4 T cells. The CSP stimulated illustrative sample from an RTS,S/AS01_E_ vaccinated individual shows production of IL-2, TNF-α, and IFN-γ by CD4 T cells.

Intracellular cytokine staining (ICS) was used to assess cell-mediated immune responses, using an adaptation of previously described methods [24]. Immediately after blood collection in Lithium-heparin tubes, whole blood samples were stimulated *in vitro* with a pool of 15-mer peptides overlapping by 11 amino acids and covering the CSP antigen in RTS,S (1.25 µg/ml), medium (negative control) or phytohemagglutin A (PHA, positive control), in the presence of anti-CD28 and anti-CD49d antibodies (BD Biosciences, Belgium). After 2 hours of incubation at 37°C, Brefeldin A (BD Biosciences) was added, and samples were incubated overnight. Red blood cells were then lysed and remaining cells were washed, fixed and frozen at −80°C until subsequent analysis. After thawing, cells were washed and stained using peridinin-chlorophyll (PerCP)-conjugated anti-CD4 (BD Biosciences) and allophycocyanin (APC)-H7 conjugated anti-CD8 antibodies (BD Biosciences). Cells were then fixed and permeabilized with the Cytofix/Cytoperm buffer kit (Pharmingen), and stained with APC conjugated anti-IL-2 (Pharmingen), fluorescein-isothiocyanate (FITC)-conjugated anti-IFN-γ (Pharmingen), phycoerythrin (PE) cyanin-7 (Cy7)-conjugated anti-TNF-α (Pharmingen) and PE-conjugated anti-CD154 (CD40L) (Pharmingen). Cells were washed, re-suspended in fetal-calf-serum (FCS)-containing phosphate buffered saline (PBS) and analyzed on a BD™ LSR II flow cytometer (BD Biosciences). Analysis was performed using BD™ Diva software (BD Biosciences). The ICS results were expressed as the number of CSP-specific CD4/CD8 T cells expressing IL-2, IFN-γ, TNF-α or CD40L per million CD4/CD8 T cells. The gating strategy and an illustrative example of cytokine response are shown in Figure 2. The analysis of CD4 T cell polyfunctionality was conducted using FlowJo software (Tree Star Inc).

### Statistical analysis

The analysis of CMI responses was performed on the ATP cohort for immunogenicity. The frequency of CSP-specific CD4, CD8 T cells expressing at least CD40L, IFN-γ, IL-2 or TNF-α was summarized for each group at peak (one month post final dose) and at month 19. Descriptive statistics (mean, median, quartiles) were tabulated by group and, within schedules, a comparison of adjuvants was made using Wilcoxon Rank Sum test. The between schedule comparison and the RTS,S/AS01_E_ versus rabies comparison were done using the mixed model procedure adjusted for multiple comparisons using the Bonferroni method to correct for type I error. A responder was defined as a subject with an outcome that was equal or greater than the geometric mean + 3 standard deviations (on the log 10 scale) of background stimulation for each cytokine. Spearman rank correlations were used to assess the correlations between the log of CSP-specific CD4 IL-2 and CD4 TNF-α responses for each schedule and both vaccine formulations with antibody levels at peak (one month post final dose) and at month 19.

## Results

### Detection of RTS,S/AS01_E_-induced CSP-specific CD4 T cell responses in infants and young children

**Figure 3:**
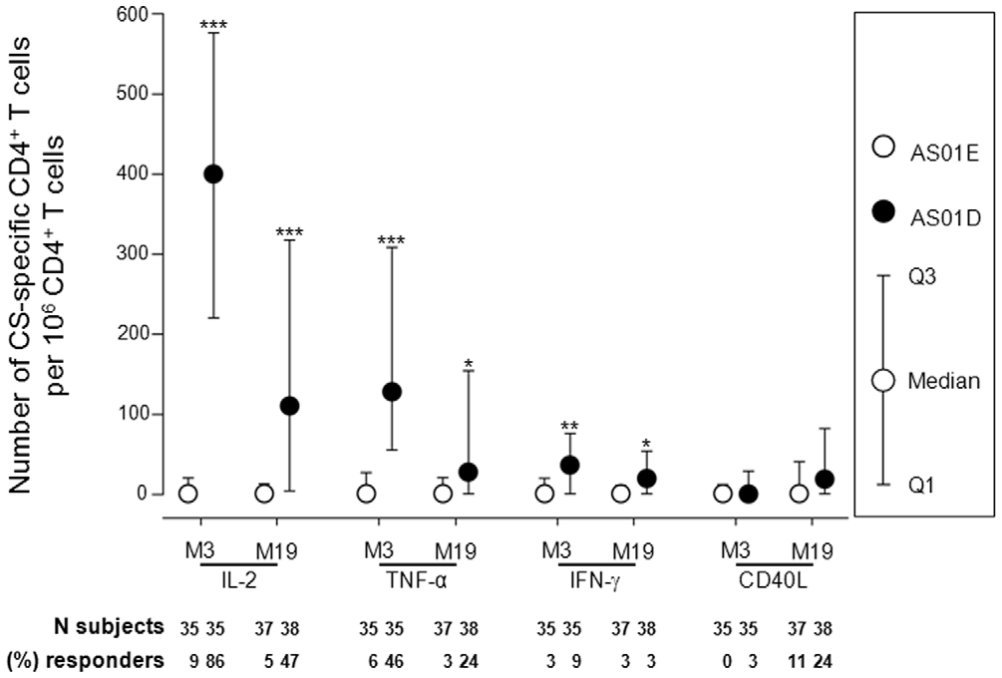
Frequency of CSP-specific CD4 T cells expressing at least IL-2, TNF-α or IFN-γ. CSP-specific CD4 T cell responses in infants and children aged 5–17 months from Kintampo, vaccinated with RTS,S/AS01_E_ or rabies vaccine according to a 0,1,2-month immunization schedule. Results are expressed as the median (with Q1 and Q3) number of CSP-specific CD4 T cells per 10^6^ CD4 T cells. The number of subjects per group and percentage responders (defined as a response that was equal or greater than the geometric mean + 3 standard deviations (on the log 10 scale) of background stimulation) is indicated. P-values were calculated using the Wilcoxon Rank Sum test. *** P<0.001, ** P<0.01, * P<0.05.

As a benchmark, the RTS,S/AS01_E_ T cell response was first compared to the response seen in the rabies group. Only data from Kintampo are presented here, as rabies vaccine was not administered in Agogo. Figure 3 presents the frequency of CSP-specific CD4 T cells expressing at least IL-2, TNF-α or IFN-γ. Compared to the rabies group, responses are modestly but significantly higher in the RTS,S/AS01_E_ vaccinated group at peak (one month post last dose) and at month 19 of the study. There was no significant difference in the expression of CD40L between both groups at either time point. No vaccine-induced CSP-specific CD8 T cell responses were detected (data not shown).

**Figure 4:**
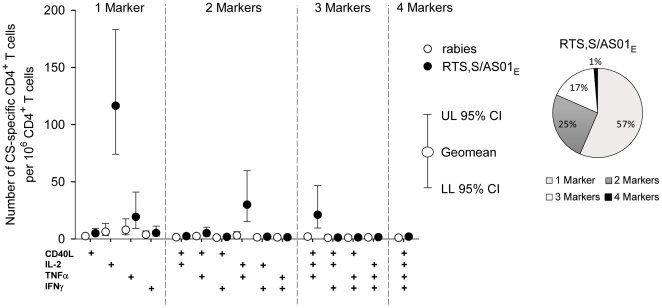
Polyfunctional profiles of CSP-specific CD4 T cells one month post last immunization. Polyfunctional profiles of CSP-specific CD4 T cells expressing any combination of immune markers among IL-2, TNF-α, IFN-γ, and CD40L in infants and children aged 5–17 months from Kintampo, vaccinated with RTS,S/AS01_E_ or rabies vaccine according to a 0,1,2-month immunization schedule. Data are represented as background subtracted geometric mean number of CSP-specific CD4 T cells expressing any combination of IL-2, TNF-α, IFN-γ, and/or CD40L per 10^6^ CD4 T cells, with 95% CI (A). The pie chart represents the proportion of CSP-specific CD4 T cells expressing 1, 2, 3 or 4 immune markers amongst IL-2, TNF-α, IFN-γ, and CD40L from RTS,S/AS01_E_ recipients (B).

A more detailed characterization of cytokine expression by CSP-specific CD4 T cells in the RTS,S/AS01_E_ vaccinated children from Kintampo showed that at one month post third vaccination the majority of CSP-specific CD4 T cells were expressing IL-2 only. Polyfunctional CSP-specific CD4 T cells were essentially IL-2^+^ TNF-α^+^ CD4 T cells and to a lesser extent CD40L^+^ IL-2^+^ TNF-α^+^ CD4 T cells (Figure 4).

### Comparison of CSP-specific CD4 T cell responses induced by RTS,S/AS01_E_ and RTS,S/AS02_D_

For the comparison of CSP-specific CD4 T cell responses induced by RTS,S/AS01_E_ and RTS,S/AS02_D_, data from the two study centers for the 0,1- and 0,1,7-month schedules were pooled. Data for the 0,1,2-month schedule are not shown as RTS,S/AS02_D_ was not administered on a 0,1,2-month schedule in Kintampo.

When administered in a three dose regimen following a 0,1,7-month schedule, RTS,S/AS01_E_ induced higher CSP-specific IFN-γ^+^ CD4 T cell responses as compared to RTS,S/AS02_D_ one month after the last vaccination, but not at month 19. The frequency of IL-2^+^ and TNF-α^+^ CD4 T cells was greater in RTS,S/AS01_E_ recipients than in RTS,S/AS02_D_ recipients at month 19, but not one month after vaccination (Table 1).

**Table 1:** **Adjuvant comparison;** CSP-specific CD4 T cell responses induced by RTS,S/AS01_E_ and RTS,S/AS02_D_ administered at 0,1- and 0,1,7-months (data pooled for both study sites) at peak (one month post last vaccination) and at study end (month 19).

			RTS,S/AS01_E_		RTS,S/AS02_D_		
Vaccine schedule	Timepoint	Marker	N (%)	M (Q1–Q3)	N (%)	M (Q1–Q3)	p-value
**0,1**	**M2**	**IL-2**	77 (52)	133 (20–391)	80 (45)	86 (24–374)	0.71
		**TNF-α**	77 (21)	38 (1–132)	80 (13)	26 (1–135)	0.62
		**IFN-γ**	77 (0)	1 (1–20)	80 (0)	1 (1–14)	0.1
		**CD40L**	77 (9)	1 (1–24)	80 (8)	1 (1–26)	0.95
	**M19**	**IL-2**	73 (19)	42 (1–96)	75 (27)	48 (1–151)	0.99
		**TNF-α**	73 (10)	14 (1–57)	75 (9)	18 (1–65)	0.9
		**IFN-γ**	73 (1)	1 (1–24)	75 (1)	1 (1–15)	0.89
		**CD40L**	73 (11)	1 (1–39)	75 (20)	29 (1–77)	**0.0012**
**0,1,7**	**M8**	**IL-2**	70 (76)	305 (68–961)	73 (71)	186 (43–852)	0.59
		**TNF-α**	70 (76)	187 (57–667)	73 (74)	162 (53–439)	0.68
		**IFN-γ**	70 (43)	57 (1–115)	73 (27)	20 (1–79)	**0.013**
		**CD40L**	70 (61)	156 (25–417)	73 (58)	119 (35–235)	0.37
	**M19**	**IL-2**	73 (53)	171 (23–365)	70 (36)	56 (1–210)	**0.0083**
		**TNF-α**	73 (30)	70 (14–211)	70 (17)	36 (1–85)	**0.015**
		**IFN-γ**	73 (1)	13 (1–44)	70 (3)	12 (1–27)	0.35
		**CD40L**	73 (32)	27 (1–116)	70 (24)	15 (1–79)	0.34

N (%): Number of subjects per group and percentage responders (defined as a response ≥ geometric mean + 3 standard deviations (on the log 10 scale) of background stimulation).

M (Q1–Q3): Results are expressed as the median (Q1 and Q3) number of CSP-specific CD4 T cells per 10^6^ CD4 T cells.P-values for comparison of RTS,S/AS01_E_ and RTS,S/AS02_D_ were calculated using the mixed model procedure adjusted for multiple comparison using the Bonferroni method to correct for type I error.

When considering the two dose regimen (0,1-month), no marked differences in the CSP-specific T cell response induced by the two vaccine formulations were seen, other than a higher frequency of CD40L^+^ CD4 T cell at month 19 after RTS,S/AS02_D_ vaccination.

### Comparison of CSP-specific CD4 T cell responses between vaccination schedules

An important objective of this study was the comparison of different vaccination schedules compatible with integration into the existing EPI vaccination program. In a previous report we have shown that anti-CSP antibody titers were higher in groups vaccinated with RTS,S/AS01_E_ using a three-dose rather than two-dose regimen [22]. CD4 T cell responses following vaccination according to different vaccination schedules with RTS,S/AS01_E_ (the formulation evaluated according to all 3 schedules in both study centers), are presented in Table 2. When considering responses one month after the last vaccination, no substantial differences were observed between a 0,1-month vaccination schedule and a 0,1,2month vaccination schedule, but compared to both other vaccination schedules, the 0,1,7-month schedule induced higher CD4 T cell responses. At month 19 however, only the TNF-α response was still higher in the 0,1,7-month schedule as compared to the 0,1-month schedule. Similar results were observed for the RTS,S/AS02_D_ formulation, when considering data from Agogo only (where RTS,S/AS02_D_ was evaluated according to all 3 vaccine schedules, data not shown).

**Table 2:** **Schedule comparison;** CSP-specific CD4 T cell responses induced by RTS,S/AS01_E_ administered at 0,1-, 0,1,2- or 0,1,7-months (data pooled over both study sites) at peak (one month post last vaccination) and at study end (month 19).

Timepoint	Marker	0,1 scheduleMedian (Q1–Q3)	0,1,2 scheduleMedian (Q1–Q3)	0,1,7 scheduleMedian (Q1–Q3)	p-value0,1 vs 0,1,2	p-value0,1 vs 0,1,7	p-value0,1,2 vs 0,1,7
Peak	**IL-2**	133 (20–391)	83 (1–372)	305 (68–961)	1	0.32	**0.031**
	**TNF-α**	38 (1–132)	35 (1–136)	187 (57–667)	1	**0.0025**	**0.0001**
	**IFN-γ**	1 (1–20)	1 (1–37)	57 (1–115)	0.7	**<0.0001**	**<0.0001**
	**CD40L**	1 (1–24)	1 (1–22)	156 (25–417)	1	**<0.0001**	**<0.0001**
Month 19	**IL-2**	42 (1–96)	79 (1–256)	171 (23–365)	1	0.072	0.89
	**TNF-α**	14 (1–57)	32 (1–104)	70 (14–211)	1	**0.0028**	0.46
	**IFN-γ**	1 (1–24)	1 (1–40)	13 (1–44)	1	0.24	1
	**CD40L**	1 (1–39)	16 (1–72)	27 (1–116)	0.84	0.058	1

M (Q1–Q3): Results are expressed as the median (Q1 and Q3) number of CSP-specific CD4 T cells per 10^6^ CD4 T cells.

P-values: Comparison were done using the mixed model procedure adjusted for multiple comparison using the Bonferroni method to correct for type I error.

### Relationship between CSP-specific CD4 T cell responses and anti-CSP antibody titers

As IL-2 and TNF-α were the cytokines most clearly activated following antigen re-stimulation of whole blood from RTS,S/AS01_E_ or RTS,S/AS02_D_ vaccinated infants and young children, the associations between CSP-specific IL-2^+^ and TNF-α^+^ CD4 T cell responses and the CSP-specific antibody responses were investigated using Spearman rank correlation index. As presented in detail in Table 3, weak but statistically significant or borderline correlations were found between peak IL-2^+^ and TNF^+^ CD4 T cell responses and serum anti-CSP antibodies at the time of the peak response and at month 19 in children in the RTS,S/AS01_E_ 0,1,2-month schedule and in the RTS,S/AS02_D_ 0,1,7-month schedule groups.

**Table 3:** Evaluation of the correlation between CSP-specific IL-2^+^ and TNF-α^+^ CD4 T cell responses one month post last vaccination (peak) with anti-CSP antibody titers at peak and at month 19 (analysis of data pooled over both study sites).

	RTS,S/AS01_E_			RTS,S/AS02_D_		
**Analysis of correlation between peak CD4 T cell response and peak antibody response**						
**Schedule**	**0,1**	**0,1,2**	**0,1,7**	**0,1**	**0,1,2**	**0,1,7**
**IL-2**	0.07125(p = 0.54)	0.25574**(p = 0.028)**	0.07924(p = 0.51)	0.20485(p = 0.068)	−0.11734(p = 0.47)	0.28147**(p = 0.016)**
**TNF-α**	0.27930**(p = 0.014)**	0.22607(p = 0.053)	0.01716(p = 0.89)	0.18516**(p = 0.1)**	0.05244(p = 0.75)	0.35847**(p = 0.0018)**
**Analysis of correlation between peak CD4 T cell response and M19 antibody response**						
**IL-2**	0.32018**(p = 0.0051)**	0.37385**(p = 0.0011)**	0.18822(p = 0.12)	0.28242**(p = 0.015)**	0.15637(p = 0.34)	0.27181**(p = 0.022)**
**TNF-α**	0.35850**(p = 0.0016)**	0.31764**(p = 0.0062)**	0.16431(p = 0.17)	0.24323**(p = 0.037)**	0.25317(p = 0.12)	0.32417**(p = 0.0058)**

The relationship between CSP-specific CD4 T cell response and CSP-specific antibody level was analyzed using Spearman's rank correlation and associated p-values are shown.

## Discussion

In this study we have investigated antigen-specific T cell responses in infants and young children aged 5–17 months, vaccinated with RTS,S/AS01_E_ or RTS,S/AS02_D_ according to three different immunization schedules. The rationale for investigating cellular responses to the CSP antigen in field studies is based on a growing body of evidence suggesting an important role of vaccine induced T cell responses targeting the pre-erythrocytic stage of malaria infection in the protection provided. This was initially demonstrated in experimental animal models [20]. In human adults, an association between vaccine efficacy and both anti-CSP humoral and CD4 T cell response was shown in individuals vaccinated with RTS,S/AS01 and RTS,S/AS02 who then went through an experimental sporozoite challenge [5]. Other vaccine candidates target protection from CD8 responses targeting pre-erythrocytic antigens [25]

This study has shown that RTS,S/AS01_E_ vaccination of infants and children induces CSP-specific CD4 T cells expressing IL-2, TNF-α or IFN-γ. These results are in line with previously shown RTS,S/AS01 induced responses in adults [5]. Quantitative differences and the absence of CD40-L induction may be related to physiologic differences, or to the fact that the pediatric assay uses whole blood antigen stimulation while PBMC were used in adults. As in most other RTS,S vaccination studies, CSP-specific CD8 T cells were not detected, but it is possible that they may not be detected in peripheral blood one month after vaccination, while still playing a role *in vivo*.

Whether activated CSP-specific CD4 T cells have a direct anti-parasite effector role, or whether they act indirectly by supporting other effector functions remains to be demonstrated. A direct role of activated CD4 T cells against infected liver cells is possible, as several cell types present in the liver, such as Kupffer cells, liver dendritic cells, endothelial cells and hepatocytes themselves can express MHC Class II molecules necessary for antigen presentation to CD4 T cells [20]. CD4 T cells, through the expression of TNF-α and IFN-γ, could contribute to the elimination of intracellular *Plasmodia* [18], [20], [26], or through other yet uncharacterized effector mechanism(s). CD4-derived IL-2 could also help NK or CD8 T cells to clear parasites as has been shown in blood-stage infection [27].

Whether or not they display intrinsic protective effector functions, CD4 T cells are likely to contribute to antibody production. In this study, weak but significant correlations were found between the anti-CSP CD4 T cell and antibody responses. This observation is in line with the well known helper T cell function, providing help to B cells, promoting antibody class switch, affinity maturation and induction of memory B cells [28], [29], thus potentially contributing to antibody-mediated protection [30]. IL-2 production by CSP-specific CD4 T cells could play an important role in the maintenance of circulating anti-CSP antibodies [31].

This study and previous trials in adults and children showed that the AS01 formulated RTS,S vaccine induces higher anti-CSP antibody responses than the AS02 formulation [5], [22], [32], [33]. In a trial in malaria-naïve American adults, superior T cell responses, humoral responses and a trend towards higher protection against *P. falciparum* infection in the experimental challenge model were also shown after RTS,S/AS01 vaccination, as compared to RTS,S/AS02 [5]. In the study described here, the adjuvant comparison generally favored RTS,S/AS01_E_ when considering the IFN-γ response at one month after the last vaccination and the IL-2 and TNF-α response at study end in the 0,1,7-month schedule. This was not seen when comparing the vaccine formulations in the 0,1-month schedule. Overall, when considering the humoral [22] and cellular immunogenicity data, the results from this trial are supportive of the selection of RTS,S/AS01_E_ for further Phase III evaluation.

For a new public health intervention in Sub-Saharan Africa, implementation into an existing delivery program is a key success factor. For a new vaccine, it is important that the immunization schedule be compatible with the existing EPI program. In this study, three immunization schedules were evaluated: a 0,1,2-month schedule that could be delivered together with the diphtheria, tetanus, pertussis, *Haemophilus influenza* type b and hepatitis B vaccines (DTP-HepB/Hib), a 0,1-month schedule which would have the advantage of only two doses, and a 0,1,7-month schedule with a delayed third dose that could be administered with the measles vaccination in the infant EPI program.

A study of 1 to 4 year old children in Gabon showed that anti-CSP antibody levels after three doses of RTS,S/AS01_E_ or RTS,S/AS02_D_ were higher than those obtained after two doses [32]. This was confirmed in the present trial, as presented in the initial report [22], and in a study of RTS,S/AS01_E_ administered to infants together with EPI vaccines [15]. A three dose immunization schedule was therefore selected for further RTS,S/AS01_E_ evaluation.

In the present study, although the humoral responses induced by a 0,1,2- and a 0,1,7-month schedule were comparable in terms of area under the curve for anti-CSP antibody titer evolution over time, the peak (one month post last dose) antibody titer following a 0,1,2-month immunization schedule was superior to the peak following a 0,1,7-month immunization schedule [22]. When considering the CD4 T cell response as reported here, no differences were detected between children who received RTS,S/AS01_E_ on a 0,1- or 0,1,2-month schedule, but the 0,1,7-month induced higher responses one month after the last vaccination. The differences between the 0,1,2- and the 0,1,7-month schedules were no longer detected at month 19. The physiological basis for better peak antibody responses with a 0,1,2- over a 0,1,7-month schedule, but opposite observation when considering CD4 T cell responses, are unclear. Delaying the last immunization dose is classically described as being favorable to immunogenicity in young children [34], as seen here with CD4 T cell responses but not antibody responses. The clinical significance of these observations remains unclear. Ongoing studies are evaluating respective vaccine efficacy of a 0,1,2- and a 0,1,7-month RTS,S/AS01_E_ infant immunization schedule.

Altogether, the CMI data reported in this study combined with the anti-CSP antibody responses in children described previously [15], [22], [32] support the ongoing Phase III evaluation of protective efficacy and immunogenicity of RTS,S/AS01_E_ administered using a three dose regimen.

## Supporting Information

Checklist S1 CONSORT Checklist.
(DOC)

Protocol S1 Trial Protocol.
(TXT)
